# Clinical Value of Asymmetrical Dimethylarginine Detection in Patients with Connective Tissue Disease-Associated Pulmonary Arterial Hypertension

**DOI:** 10.1155/2019/3741909

**Published:** 2019-08-14

**Authors:** Juan Liu, Qiang Fu, Lili Jiang, Youlian Wang

**Affiliations:** Department of Rheumatology, Jiangxi Provincial People's Hospital, Nanchang 330006, China

## Abstract

**Objective:**

To evaluate the clinical value of serum asymmetrical dimethylarginine (ADMA) in patients with connective tissue disease- (CTD-) associated pulmonary arterial hypertension (PAH).

**Methods:**

88 patients with CTD were recruited between December 2017 and August 2018 in Jiangxi Provincial People's Hospital. Patients were further divided into two groups: CTD-without PAH (*n* = 45 cases) and CTD-with PAH (*n* = 43 cases), according to the pulmonary systolic blood pressure measured by echocardiography. 40 healthy controls were also included (*n* = 40 cases). The clinical data, including laboratory examinations, echocardiographic measurements, pulmonary function, and serum ADMA levels determined by enzyme-linked immunosorbent assay, (ELISA) were collected. The correlation between ADMA levels and the occurrence of PAH, pulmonary function, and other laboratory indexes in CTD patients were analyzed. Statistical analyses were performed by SPSS (version 23); *P* < 0.05 was considered statistically significant.

**Results:**

The serum levels of ADMA in the CTD-PAH group were significantly higher than those of the CTD-without PAH group and healthy control group (*P* < 0.05); the serum ADMA levels were (0.706 ± 0.153 *μ*mol/L), (1.015 ± 0.122 *μ*mol/L), and (0.661 ± 0.113 *μ*mol/L), respectively. There was no significant difference between the CTD-without PAH group and healthy control group (*P* < 0.05). Correlation analysis showed that serum ADMA levels were positively correlated with sPAP and NT-proBNP and negatively correlated with DLCO% (*r* = 0.802, 0.475, −0.585, *P* < 0.001). Multivariate analysis indicated that elevated serum ADMA levels increased the risk for the appearance of PAH in CTD patients (*OR* = 57.460, *P* < 0.001). Using the receiver operating characteristic (ROC) curve analysis, at the cutoff level of 0.810 *μ*mol/L, ADMA showed good diagnostic efficacy as follows: sensitivity was 97.7%, specificity was 75.6%, and the area under the curve (AUC) was 0.947 (*P* < 0.001).

**Conclusion:**

Increased ADMA levels are independently associated with the presence and severity of PAH in CTD patients. The levels of ADMA in the serum may contribute to be a noninvasive indicator for early diagnosis of CTD-with PAH patients.

## 1. Introduction

Connective tissue disease (CTD) is an autoimmune disease based on chronic inflammation of blood vessels and connective tissue, which can involve immune injury and dysfunction in multiple systems of the whole body. Pulmonary arterial hypertension (PAH) is a common complication when CTD involves the lungs and is one of the important factors of death in CTD. PAH is a hemodynamic and pathophysiological state in which the pulmonary artery pressure rises above a certain cutoff value. It is a progressive disease caused by a remodeling of precapillary arterioles that leads to a gradual increase in pulmonary vascular resistance and right ventricular failure. PAH can be an independent disease or a complication.

Various types of CTD may be complicated with PAH; the main clinical manifestations are cough, chest tightness, palpitation, decreased mobility, dyspnea, and finally right heart failure. PAH is easily overlooked, because its early clinical manifestations are not characteristic, and some symptoms of CTD itself overlap with the clinical manifestations of PAH, which all lead to the complication and severity of the disease. So the PAH is an important factor affecting the prognosis of CTD. The diagnosis and severity evaluation of PAH has become an important part of the treatment and prognosis evaluation of CTD. Studies have shown that early screening, diagnosis, and treatment can effectively improve the prognosis of CTD-PAH patient and 1-year and 3-year survival rates can be increased to 94% and 73%, respectively [1].

At present, pulmonary artery pressure ≥25 mmHg measured by right cardiac catheterization (RHC) in the resting state is the gold standard for the diagnosis of PAH [2].

However, due to the limitation of invasive, high cost, and demand for high medical technology, RHC is not suitable for repeated operation to assess the condition and treatment effect and it cannot be a good diagnosis and follow-up index. Echocardiography is an important noninvasive screening method for PAH, which is in good agreement with right cardiac catheterization, but it is difficult to standardize because of the high requirements for technical level of operators. Therefore, the search for simple detection methods to judge the occurrence and development of CTD-PAH is one of the research hotspots. If one or more substances that play a key role in the pathogenesis of CTD-PAH are identified, it will bring more choices and hopes for the diagnosis and treatment of CTD-PAH.

We have searched the literature and found that asymmetric dimethylarginine (ADMA) is an inhibitor of endogenous nitric oxide synthase (NOS) that can affect endothelial function and resistance of pulmonary vessels by affecting the production of nitric oxide (NO), which is a diastolic vascular substance. It has been reported in the literature that ADMA can be used as a noninvasive screening indicator for early identification of multiple types of PAH such as congenital heart disease with PAH, idiopathic pulmonary hypertension (IPAH), and chronic thromboembolic pulmonary hypertension [3–5], while the clinical value of ADMA in CTD-PAH is rarely reported. This study aims to explore the significance of ADMA in the occurrence and development of CTD-PAH, to explore its value in the diagnosis of CTD-PAH.

## 2. Subjects and Methods

### 2.1. Study Population

We performed a retrospective cohort study including 88 newly diagnosed CTD patients from the hospitalized patients in rheumatology and immunology department of our hospital (17 males and 71 females). And 40 healthy adults were recruited as a control group. All patients fulfilled, respectively, 2010 ACR/EULAR rheumatoid arthritis (RA) classification criteria, 1997 diagnostic criteria for systemic lupus erythematosus (SLE), 2016 ACR/EULAR primary Sjogren's syndrome (Pss), the classification standard of 2013 ACR/EULAR systemic sclerosis (SSc), and 1975 Bohan's diagnostic table for dermatomyositis/polymyositis (DM/PM). We excluded PAH caused by drug or toxicant, congenital heart disease, portal hypertension, HIV infection, and IPAH, as well as other forms of PAH, such as chronic thromboembolic pulmonary hypertension and PAH caused by left-heart disease, pulmonary disease, or hypoxia. Patients with coronary heart disease, myocardial infarction, atrial fibrillation, rheumatic heart disease, severe liver and kidney dysfunction, pregnancy, or malignancy would also be excluded. All included subjects were older than 18 years old. The study was approved by the Jiangxi Provincial People's Hospital Clinical Research Ethics Committee, and the subjects were informed and signed informed consent.

### 2.2. Clinical and Laboratory Characteristics

Demographic characteristics of all subjects, including gender and age were recorded.

We collected the clinical features including disease duration, Raynaud's phenomenon, comorbidities such as Chest tightness and cough, and laboratory examinations of all CTD patients.

### 2.3. Echocardiography

Examination of echocardiography (Philips EPIQ5) was performed on all permitted objects by two high-grade ultrasound physicians with five years of work experience, who did not know the patient's condition. From the apical 4-chamber view, continuous-wave Doppler echocardiography was used to assess the peak tricuspid regurgitant velocity. The right ventricular to right atrial systolic pressure gradient was calculated using a modified Bernoulli equation: 4 × (tricuspid regurgitant jet velocity) 2. SPAP was then estimated by adding that pressure gradient to the mean right atrial pressure. According to the ACCF/AHA 2009 Expert Consensus Document on Pulmonary Hypertension, PAH was defined as an sPAP value ≥ 40 mmHg in our study and all included subjects with PAH could be further divided into the mild pulmonary hypertension group (sPAP ≤ 50 mmHg) and moderate-to-severe pulmonary hypertension group (sPAP > 50 mmHg), according to the severity of sPAP [6].

### 2.4. Pulmonary Function

Pulmonary function tests were performed in all enrolled patients (Ganshorn Power Cube LF 8.5M RC31 pulmonary function tester). The main research indicators were the percentage of vital capacity (VC%), the forced vital capacity as a percentage of predicted value (FVC%), the ratio of forced expiratory volume to forced vital capacity in seconds (one second rate, FEV1.0/FVC), residual gas percentage as a percentage of predicted value (RV%), and carbon monoxide diffusion as a percentage of predicted value (DLco%).

### 2.5. Measurements and Calculation

Blood samples for measurement of serum ADMA concentrations were naturally coagulated at room temperature for 20 minutes and centrifuged for about 20 minutes (3000 rpm), and the supernatants were stored in 1 mL aliquots at −80°C until further use. Concentration of ADMA was measured in serum samples by using an enzyme immunoassay ELISA kit provided by Shanghai Renjie Biotechnology Co., Ltd. The whole detection process was completed in strict accordance with the operating instructions. The absorbance (OD) was measured at 450 nm by using a Microplate reader, and each sample was duplicated.

### 2.6. Statistical Analysis

Statistical calculations were performed using SPSS, version 23. We used descriptive statistics expressed as numbers (percentages) in categorical variables, whereas continuous variables were expressed as mean ± standard deviation ($\bar{x}\pm s$) and the median with interquartile range (M(QR)) values in accordance with their distribution. The differences between continuous variables were compared by Student's *t*-test or the Mann–Whitney test according to their distribution. Significant differences between the categorical variables were evaluated using the chi-square test. We performed univariate and multivariate analyses using nonconditional logistic regression to calculate independent association of the presence of CTD-with PAH with levels of serum ADMA. The correlation between two continuous variables was evaluated by Spearman's rank correlation coefficient. Also, the receiver operating characteristic (ROC) curve was used to establish the sensitivity and specificity of ADMA for the diagnosis of the CTD-with PAH. The significance level was set at *P* < 0.05.

## 3. Results

### 3.1. Participant Characteristics

A total of 88 CTD patients and 40 healthy adults were enrolled in this study and divided into three groups, including CTD-without PAH (*n* = 45 cases), CTD-with PAH (*n* = 43 cases), and healthy control (*n* = 40 cases). There was no significant difference in age and sex among the three groups (*P* > 0.05). The comparison between CTD-without PAH group and CTD-with PAH group showed that no significant difference in the average course of disease (12 (4.5, 72) versus 24 (6, 84) months; *P* > 0.05). The recruited CTD patients included 31 RA patients, 28 SLE patients, 3 pSS patients, 15 SSc patients, and 11 DM/PM patients. No significant difference in disease distribution between the CTD-without PAH group and CTD-with PAH group was found (*P* > 0.05).

### 3.2. Clinical Symptoms and Auxiliary Examinations

Comparisons of the clinical manifestations and laboratory examination indexes between CTD-without PAH and CTD-with PAH patients were summarized in Table 1. Also, parameters of echocardiography and pulmonary function were compared in Table 2.

No significant differences were found between the two groups regarding ESR, lactate dehydrogenase, complement, immunoglobulin, and D-dimer (all *P* > 0.05, Table 1). The serum levels of C-reactive protein, average platelet volume, total bilirubin, serum uric acid, homocysteine, ferritin, and N-terminal brain natriuretic peptide precursor (NT-proBNP) in CTD-with PAH patients were distinctly higher than those in CTD-without PAH patients (all *P* < 0.05, Table 1).

SPAP in the CTD-without PAH group and CTD-with PAH group were 23.0 (18.0, 28.0) mmHg and 51.0 (46.0, 54.0) mmHg, respectively. The pulmonary function indexes such as VC%, FVC%, and DLCO% in CTD-with PAH patients were obviously lower than those in CTD-without PAH patients (all *P* < 0.05, Table 2), while the difference of ejection fraction, cardiac output, FEV1/FVC, and RV% between groups was not clear (all *P* > 0.05, Table 2).

### 3.3. Serum Level of ADMA

**Figure 1:**
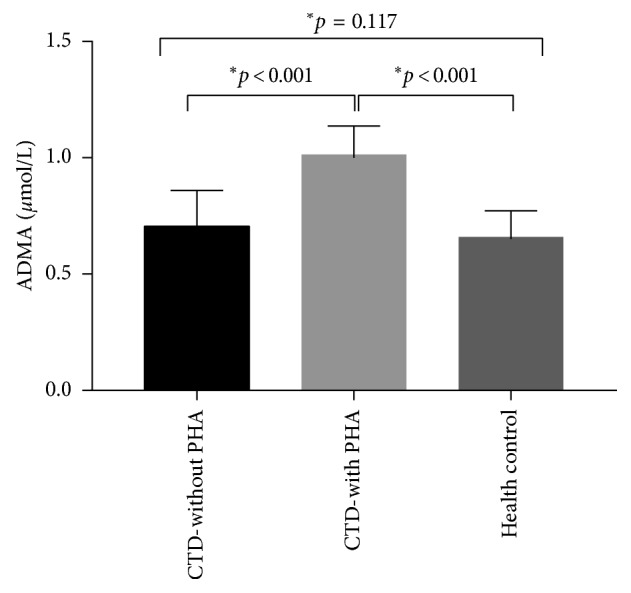
Comparison of serum ADMA levels among three groups. Serum ADMA levels were measured in CTD-without PAH group, CTD-with PAH group, and healthy control group. Data were expressed as mean ± SD. ^*∗*^*P* < 0.001 compared with the CTD-with PAH group.

The serum ADMA levels of patients in CTD-without PAH, CTD-with PAH, and healthy control were (0.706 ± 0.153 *μ*mol/L), (1.015 ± 0.122 *μ*mol/L), and (0.661 ± 0.113 *μ*mol/L), respectively.  Figure 1 showed that the difference of serum ADMA levels among CTD-without PAH, CTD-with PAH, and healthy control group were statistically significant (*P* < 0.05), whereas there was no significant difference in ADMA concentrations between the CTD-without PAH group and healthy control group (*P* > 0.05).

#### 3.3.1. Comparison of Serum ADMA Levels in CTD Patients with Different Severities of PAH

According to the level of sPAP, all included patients with PAH were divided into the mild group (sPAP < 50 mmHg) and moderate-to-severe group (sPAP > 50 mmHg). Subgroup analysis showed that the serum ADMA levels of CTD with moderate-to-severe PAH patients were significantly higher compared to CTD with mild PAH patients (1.070 (1.020, 1.170) vs 0.915 (0.860, 1.045) *μ*mol/L, *P* < 0.05, Table 3).

#### 3.3.2. Comparison of Serum ADMA Levels among Different Types of CTD

CTD-with PAH patients enrolled in this study included 16 patients with RA, 12 patients with SLE, 8 patients with SSc, and 5 patients with DM/PM. Serum ADMA levels among various diseases were 1.025 (0.900, 1.115) *μ*mol/L, 1.055 (0.878, 1.150) *μ*mol/L, 1.045 (1.003, 1.170) *μ*mol/L, and 0.990 (0, 1.090) *μ*mol/L, respectively. There was no significant differences found among different types of CTD (*Z* = 1.204, *P* > 0.05) (Pss with PAH was not included in the analysis because of few samples).

### 3.4. Correlation Analysis

**Figure 2:**
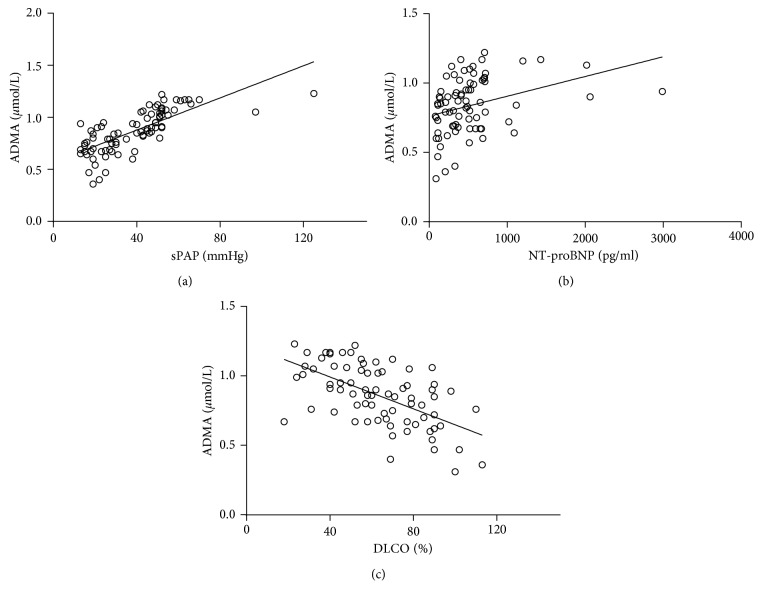
Association of serum ADMA levels with sPAP, NT-proBNP, and DlCO%. Scatter plots of the relationship in serum ADMA concentration. A: sPAP (*r* = 0.802, *P* < 0.05), B: NT-proBNP (*r* = 0.475, *P* < 0.05), and C: DLCO% (*r* = −0.585, *P* < 0.05), in 88 CTD patients.

In spearman correlation analysis, serum ADMA levels were positively correlated with sPAP (*r* = 0.802, *P* < 0.05) and plasma NT‐proBNP levels (*r* = 0.475, *P* < 0.05) in CTD patients and negatively correlated with DLCO% (*r* = −0.585, *P* < 0.05). (Figure 2).

### 3.5. Unconditional Logistic Regression Analysis on Predictors of PAH in CTD Patients

Univariate analysis showed that in patients with CTD, higher levels of mean platelet volume, total bilirubin, serum uric acid, chest tightness, or Reynolds and serum ADMA were correlated with the occurrence of PAH (all *P* < 0.05, Table 4). Further multivariate analysis showed that high serum ADMA levels increased the risk for the appearance of PAH in CTD patients (OR = 50.348, 95% CI = 7.596–333.707, *P* < 0.05) (Table 4).

### 3.6. Diagnostic Value of Serum ADMA in CTD-with PAH

**Figure 3:**
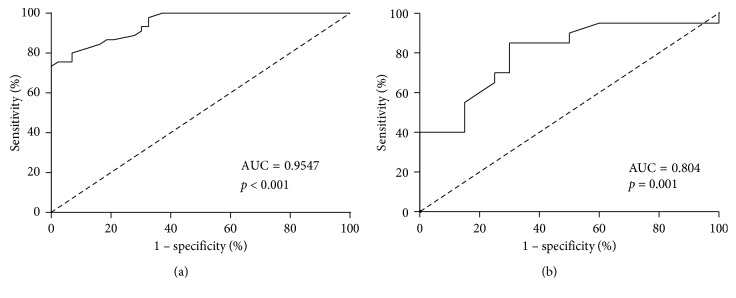
ROC curve for serum ADMA levels; ROC curve analysis evaluated optimal cutoff values of serum ADMA levels for predicting new occurrences of PAH (3(a) cutoff value = 0.810 *μ*mol/L) and moderate to severe PAH ((b) cutoff value = 0.995 *μ*mol/L) in CTD patients. ROC curve: receiver operating characteristic curve; AUC: area under the curve.

We used the ROC curve to determine the sensitivity and specificity of serum ADMA in CTD patients with PAH. Serum ADMA could predict the presence of PAH in CTD patients, with a sensitivity of 97.7% and a specificity of 75.6% at a cutoff value of 0.810 *μ*mol/L. The area under the curve was 0.947 (Figure 3(a)). Based on further analysis, the value for the serum ADMA level to detect the development of moderate-to-severe PAH in CTD patients with a sensitivity of 87.0% and a specificity of 70.0% was 0.995 *μ*mol/L. The area under the curve was 0.804 (Figure 3(b)).

## 4. Discussion

The main finding of this study was that serum ADMA levels in CTD-with PAH were significantly higher than those in CTD-without PAH patients and healthy adults. Compared with mild PAH, serum ADMA levels increased more in CTD patients with moderate to severe PAH. Moreover, in patients with CTD, serum ADMA concentrations correlated positively with predictors of severity such as NT-proBNP. Multivariate analysis showed that higher serum ADMA levels increased the risk of PAH in CTD patients. Our results demonstrate that serum ADMA levels were significantly associated with the occurrence and development of PAH in CTD patients. A number of studies have found that serum ADMA levels are elevated in multiple types of PAH patients (such as congenital heart disease with PAH, IPAH, chronic thromboembolic pulmonary hypertension, chronic obstructive pulmonary disease-related PAH, and HIV infection-related pulmonary hypertension) [3–5, 7–9] and increased ADMA concentrations are associated with increased pulmonary arterial pressure. The above researches suggest that ADMA may participate in the disease process of PAH. However, there are few reports on the clinical value of serum ADMA for CTD-with PAH, especially in China. The purpose of this study was to explore the correlation between the elevation of serum ADMA levels and the occurrence and development of PAH in patients with CTD and to explore the diagnostic value of ADMA for CTD-PAH.

The pathogenesis of CTD-PAH is not clear. It may be associated with spastic contraction of pulmonary arterioles, pulmonary vascular endothelial hyperplasia and remodeling, thrombosis in situ of lung vascular, endothelial dysfunction and disturbance of vasoactive mediators affecting pulmonary vascular pressure (such as overproduction of vasoconstrictor or reduction of vasodilator production), inflammation, autoimmunity, and other mechanisms [10, 11]. Human studies have shown that the occurrence of PAH is associated with a decrease in endogenous NO [12], which is an important physiological vasodilator produced and secreted by endothelial cells. It plays an important role in vasodilation by regulating the content of cyclic guanosine monophosphate in vascular smooth muscle and increasing intracellular calcium efflux. Moreover, NO can inhibit the thrombosis and proliferation of vascular muscle cells, which plays a major part in regulating blood flow and maintaining normal vascular wall structure [9]. It can be seen that endothelial dysfunction caused by the decrease of bioavailability of endogenous vasodilator NO may have important roles in the occurrence and development of PAH. Previous studies have suggested that the decrease of NO production in vivo is related to the acceleration of NO metabolism, the reduction of NOS substrates, and decrease of NOS activity [13]. NO is synthesized in the endothelium from the amino acid L-arginine by the action of NO synthase (NOS), which is blocked by endogenous L-arginine analogs. The most potent of these endogenous NOS inhibitors is ADMA [4], which can not only compete with L-arginine for the active site of NOS but also reduce the concentration of NO synthesis substrate by interfering with the transport of L-arginine to cells. All these lead to the blockage of NO synthesis and affect pulmonary vascular resistance.

ADMA is an endogenous NOS inhibitor, produced by methylation of arginine residues and metabolized by dimethyl-arginine dimethylaminohydrolase (DDAH). When the function of endothelial cell is impaired, oxidative stress can lead to increased ADMA production [14]. At the same time, hypoxia can induce the decrease of endothelial DDAH II expression and activity in pulmonary vascular tissue, which leads to the decrease of ADMA decomposition in the lung [5]. ADMA accumulates in the lungs through the above two metabolic pathways, resulting in the activity decline of NOS. It eventually leads to a decrease in NO production in vivo, which can inhibit vasodilation and promote the formation of PAH. The elevation of ADMA levels caused by suppressing the expression and function of DDAH II have been confirmed to be an important potential mechanism of PAH induced by monocrotaline, hypoxia-related PAH, and IPAH [5]. Experiments in mice confirmed that the metabolism of ADMA played an important role in regulating NOS activity and the increase of ADMA level was accompanied by the decrease of NOS activity [15]. Previous studies reported that the measured value of NO in exhaled gas of IPAH patients was lower than that of healthy controls and histological examination of human lung samples showed that the expression of NOS in pulmonary vessels of IPAH patients was lower than that of healthy lungs [12]. These results further support our conclusion that ADMA-NOS/NO system is closely related to the occurrence of PAH in CTD patients. Our study observed that the main abnormalities of pulmonary function in CTD-with PAH patients were restrictive ventilation dysfunction and diffusive dysfunction. It may be related to the abnormal respiratory function caused by pulmonary vascular structural changes invading the small airway and the insufficient pulmonary perfusion contributed by occlusive disease of pulmonary small vessel in patients with PAH.

From the results of our study, we found that CTD-with PAH patients were more likely to develop Raynaud's phenomenon and chest tightness than CTD-without patients. It is considered that there may be pathophysiological changes similar to Raynaud's phenomenon in patients with PAH, namely, pulmonary vasospasm, also known as pulmonary Raynaud's phenomenon. Under the stimulation of cold hypoxia, pulmonary vasospasm and contraction lead to the increase of pulmonary vascular resistance. If this situation happens repeatedly, the lung tissue will be anoxic, resulting in chest tightness, shortness of breath, and decrease of blood oxygen in patients. These research findings suggest that PAH should be screened in time for patients with the aforementioned symptoms.

We also demonstrated that the high level of serum uric acid was an important influencing factor for the presence of PAH in CTD patients, which was consistent with the conclusion of Njaman et al. [16]. It may be related to factors such as endothelial injury and inflammation, aggravation of pulmonary vasoconstriction caused by tissue hypoxia, and increased blood viscosity and raised the risk of pulmonary vascular thrombosis caused by urate crystallization attached to the vascular endothelium.

Several limitations of the present study should be mentioned. Due to the cost of examination and the willingness of the patients, it is not possible to use the RHC to screen PAH patients. However, the coincidence between the estimation of sPAP by echocardiography and RHC is acceptable, which can reflect the PAH to a certain extent. Greiner et al. [17] compared the results of echocardiography and RHC in 1695 patients and found that the correlation coefficient of sPAP values estimated by the two methods was 0.87. It was concluded that echocardiography is a reliable method for evaluating the level of sPAP.

## 5. Conclusions

In conclusion, the serum ADMA levels in CTD patients with PAH are significantly elevated and high serum ADMA levels are associated with the occurrence and severity of PAH. Also, it has good diagnostic accuracy for CTD-with PAH. Our results suggest that ADMA may be a useful biomarker for the diagnosis of PAH and related to the severity of the disease. Moreover, the detection method of the serum ADMA level is noninvasive and valuable so that it can be used as a serological index for follow-up evaluation of CTD-PAH patients. At the same time, this study also suggests that the ADMA-NOS/NO system can provide a new potential target for the treatment of CTD-PAH in the future, which is worthy of more studies. The sample size of this study is small, and the conclusion still needs to be further confirmed by a larger sample size and prospective cohort studies.

## Figures and Tables

**Table 1:** Baseline clinical data of CTD patients.

Parameters	Normal sPAP (*n* = 45)	Abnormal sPAP (*N* = 43)	*P* value
Chest tightness (*n*, %)	6 (13.3)	18 (41.9)	0.003^*∗*^
Raynaud's phenomenon (*n*, %)	6 (13.3)	14 (32.6)	0.031^*∗*^
Cough (*n*, %)	9 (20.0)	13 (30.2)	0.268
ESR ($\bar{x}\pm s$, mm/h)	24 (16, 46)	40 (19, 60)	0.199
CRP (M(QR), mg/L)	3.49 (1.95, 22.60)	11.5 (4.60, 28.05)	0.034^*∗*^
PLT ($\bar{x}\pm s$, L)	214.8 ± 71.29	177.3 ± 83.4	0.026^*∗*^
MPV (M(QR), fl)	10.6 (9.8, 11.7)	11.3 (10.4, 12.8)	0.021^*∗*^
RDW (M(QR), %)	13.4 (12.8, 14.8)	13.9 (13.2, 15.9)	0.191
TBIL (M(QR), *μ*mol/L)	9.1 (7.4, 11.3)	12.4 (9.8, 14.2)	<0.001^*∗*^
UA (M(QR), *μ*mol/L)	262.5 (230.3, 298.0)	308.0 (244.0, 439.0)	0.013^*∗*^
Hcy (M(QR), *μ*mol/L)	10.2 (7.3, 11.7)	12.5 (10.3, 15.4)	0.001^*∗*^
LDH (M(QR), U/L)	191.0 (159.8, 277.0)	204.5 (169.3, 298.8)	0.517
Ferritin (M(QR), ng/ml)	132.95 (74.55, 312.68)	224.00 (122.50, 412.40)	0.043^*∗*^
D-D (M(QR), mg/L)	0.46 (0.21, 1.60)	0.87 (0.48, 1.46)	0.065
FDP (M(QR), mg/L)	2.96 (1.13, 6.19)	4.02 (2.23, 7.99)	0.077
NT-proBNP (M(QR), pg/ml)	337.0 (144.0, 594.0)	522.0 (353.0, 713.3)	0.003^*∗*^
C3 (M(QR), g/L)	0.82 (0.61, 0.93)	0.76 (0.55, 0.99)	0.871
C4 (M(QR), g/L)	0.17 (0.12, 0.20)	0.16 (0.12, 0.23)	0.816
IgG (M(QR), g/L)	12.70 (8.82, 18.30)	12.40 (10.20, 16.40)	0.767
IgM (M(QR), g/L)	1.12 (0.57, 1.62)	0.97 (0.69, 1.71)	0.802
IgA (M(QR), g/L)	2.47 (1.54, 3.55)	2.30 (1.20, 3.34)	0.695
Autoimmune-related antibodies^a^ (*n*, %)	28 (62.2)	24 (55.8)	0.541

^a^Antinuclear antibody positive, or at least one autoantibody appears in the antinuclear antibody spectrum; ESR: erythrocyte sedimentation rate; CRP: C-reactive protein; HGB: hemoglobin; PLT: platelets; MPV: mean platelet volume; Hct: hematocrit; RDW: erythrocyte hemoglobin distribution width; TBIL: total bilirubin; UA: uric acid; Hcy: homocysteine; LDH: lactate dehydrogenase; D-D: D-dimer; FDP: fibrin degradation product; NT-proBNP: N-terminal pro-brain natriuretic peptide; IgG: immunoglobulin G; IgM: immunoglobulin M; IgA: immunoglobulin A; ^*∗*^*P* < 0.05.

**Table 2:** Comparison of echocardiography and pulmonary function in patients with CTD.

Parameters	Normal sPAP (*n* = 45)	Abnormal sPAP (*N* = 43)	*P* value
sPAP (M(QR), mmHg)	23.0 (18.0, 28.0)	51.0 (46.0, 54.0)	<0.001^*∗*^
EF (M(QR), %)	59.0 (51.0, 65.5)	60.0 (56.0, 65.0)	0.287
CO ($\bar{x}\pm s$, L)	4.2 (3.1, 4.9)	4.1 (3.7, 5.4)	0.619
VC% ($\bar{x}\pm s$, %)	83.51 ± 17.98	72.80 ± 19.05	0.012^*∗*^
FVC% ($\bar{x}\pm s$, %)	83.35 ± 17.13	73.20 ± 19.51	0.016^*∗*^
FEV1/FVC ($\bar{x}\pm s$, %)	92.36 ± 8.25	90.28 ± 9.59	0.304
RV% (M(QR), %)	99.0 (90.0, 105.0)	103.0 (96.5, 111.0)	0.063
DLCO% ($\bar{x}\pm s$, %)	72.28 ± 20.97	53.92 ± 19.37	<0.001^*∗*^

^*∗*^ *P* < 0.05; sPAP = pulmonary arterial systolic pressure; EF = ejection fraction; CO = cardiac output; VC% = percentage of vital capacity to the estimated value; FVC% = percentage of forced vital capacity to the estimated value; FEV1/FVC = forced the first second of expiratory volume/forced vital capacity; RV% = percentage of residual gas to the estimated value; DLCO% = percentage of diffusing capacity for carbon monoxide to the estimated value.

**Table 3:** ADMA of CTD patients with different degrees of PAH.

	Number	sPAP (mmHg)	ADMA (*μ*mol/L)
Mild PAH	20	45.5 (43.0, 48.5)	0.915 (0.860, 1.045)
Moderate to severe PAH	23	53.0 (52.0, 63.0)	1.070 (1.020, 1.170)

*Z* value		5.620	3.413
*P* value		<0.001^*∗*^	*P*=0.001^*∗*^

^*∗*^ *P* < 0.05.

**Table 4:** Univariate and multivariate analysis.

	Predictor	OR (95% CI)	*P* value
Univariate analysis	MPV	1.451 (1.076, 1.956)	0.015^*∗*^
	TBIL	1.196 (1.053, 1.358)	0.006^*∗*^
	UA	1.005 (1.001, 1.009)	0.014^*∗*^
	Chest tightness	4.680 (1.635, 13.395)	0.004^*∗*^
	Raynaud's phenomenon	3.138 (1.076, 9.151)	0.036^*∗*^
	ADMA	57.460 (9.357, 352.854)	<0.001^*∗*^

Multivariate analysis	UA	1.008 (1.002, 1.015)	0.015^*∗*^
	ADMA	50.348 (7.596, 333.707)	<0.001^*∗*^

^*∗*^ *P* < 0.05.

## Data Availability

The data used to support the findings of this study are restricted by the Jiangxi Provincial People's Hospital Clinical Research Ethics Committee in order to protect patient privacy. Data are available from Juan Liu (e-mail: 980614516@qq.com) for researchers who meet the criteria for access to confidential data.
